# Supplementary material for: Antiradical and Antioxidant Activity and Stimulation of Pancreatic Lipase by Extracts Obtained from Saponin-Rich Raw Materials: Experimental and In Silico Study
Source: Int J Mol Sci. 2025 Oct 22;26(21):10254. doi: 10.3390/ijms262110254 (PMC12608017; doi:10.3390/ijms262110254)
Supplement: Supplementary file 1 [file ijms-26-10254-s001.zip › ijms-3845671-supplementary.pdf]

Table S1 Equilibrium Surface tension, surface elasticity modulus, and surface viscosity modulus of rich saponins extracts obtained from some raw materials (±SD, n=3).

Sample	Plant material	Equilibrium surface tension [mN/m]	Surface elasticity modulus [mN/m]	Surface viscosity modulus [mN/m]
Ruscus aculeatus L.	rhizome	38,0±0,3	123,7±0,6	15,0±0,7
Ruscus aculeatus L. with pancreatin		37,1±0,4	133,8±7,8	36,4±2,7
Quillaja saponaria Molina	bark	34,9±0,6	25,3±1,9	11,7±1,0
Quillaja saponaria Molina with pancreatin		41,7±0,2	59,8±16,5	22,4±4,5
Gypsophila paniculata L.	root	46,1±0,4	9,2±0,2	2,8±0,8
Gypsophila paniculata L. with pancreatin		43,1±0,2	12,2±0,7	2,1±0,4
Glycyrrhiza glabra L.	root	41,9±0,1	16,3±0,2	6,3±0,1
Glycyrrhiza glabra L. with pancreatin		33,7±0,2	28,9±0,7	6,9±0,6
Primula veris L. (*Primula officinalis* L.)	root	35,5±0,5	28,3±0,2	8,6±0,1
Primula veris L. (*Primula officinalis* L.) with pancreatin		38,2±0,6	22,6±0,5	7,7±0,4
Panax ginseng C.A. Meyer	root	32,2±0,3	22,1±0,6	10,2±0,2
Panax ginseng C.A. Meyer with pancreatin		19,8±1,3	180,7±8,7	121,7±9,7
Hedera helix L.	leaves	33,0±0,2	61,5±7,7	43,1±4,0
Hedera helix L. with pancreatin		44,4±0,9	16,4±3,7	5,2±1,6
Aesculus hippocastanum L.	seeds	30,2±1,1	26,2±1,1	8,7±0,6
Aesculus hippocastanum L. with pancreatin		42,8±0,1	18,3±2,8	14,2±3,2
Trigonella foenum-graecum L.	seeds	33,6±0,4	31,4±0,8	**10,8±0,1**
Trigonella foenum-graecum L. with pancreatin		42,6±0,7	62,1±4,4	**22,2±1,3**
Sodium cholate	-	35,9±0,3	12,7±0,4	**1,0±0,3**
Sodium cholate with pancreatin		39,3±0,6	9,5±1,9	**2,1±0,4**
pancreatin	-	50,9±0,9	47,8±3,6	**10,9±1,6**

Table S2 Putative identification of the most prominent compounds in our untargeted LC-MS analysis of the extract from *Panax ginseng* roots.

Peak no.	identification	Measured m/z $[M + HCOO]^-$	Theoretical m/z $[M + HCOO]^-$	Molecular formula (neutral)	Error (Δ ppm)
**1**	ginsenoside Rg1*	845.4904	845.4904	$C_{42}H_{72}O_{14}$	0.0

**2**	ginsenoside Re	991.5470	991.5483	$C_{48}H_{82}O_{18}$	1.3
**3**	ginsenoside Rf*	845.4904	845.4904	$C_{42}H_{72}O_{14}$	0.0
**4**	ginsenoside Rb1	1153.5975	1153.6011	$C_{54}H_{92}O_{23}$	3.1
**5**	ginsenoside Rc	1123.5890	1123.5905	$C_{53}H_{90}O_{22}$	1.4
**6**	ginsenoside Rb2*	1123.5879	1123.5905	$C_{53}H_{90}O_{22}$	2.3
**7**	ginsenoside Rb3	1123.5883	1123.5905	$C_{53}H_{90}O_{22}$	2.0
**8**	ginsenoside Rd	991.5472	991.5483	$C_{48}H_{82}O_{18}$	1.1

*Identification of these compounds was additionally confirmed using reference standards

Table S3 Putative identification of the most prominent compounds in our untargeted LC-MS/MS analysis of the extract from *Aesculus hippocastani* seeds.

**Peak no.**	**identification**	**Measured m/z [M – H]⁻**	**Theoretical m/z [M – H]⁻**	**Molecular formula (neutral)**	**Major fragment ions (m/z)**	**Error (Δ ppm)**
**1**	N-[β-D-glucopyranosyl(1→2)]-β-D-xylopyranosyl-indole-3-acetic acid	498.1613	498.1617	$C_{22}H_{29}NO_{12}$	292, 130	0.8
**2**	N-[β-D-glucopyranosyl(1→3)-[β-D-glucopyranosyl(1-4)]-β-D-xylopyranosyl-indole-3-acetic acid	630.2049	630.2040	$C_{27}H_{37}NO_{16}$	468, 262, 130	1.4
**3**	N-[β-D-glucopyranosyl(1→3)]-β-D-xylopyranosyl-indole-3-acetic acid	468.1516	468.1511	$C_{21}H_{27}NO_{11}$	262, 130	0.9
**4**	Phenylethanol glycoside	475.1831	475.1821	$C_{21}H_{32}O_{12}$	205, 163	2.1
	Flavonoid	919.2359	919.2361	$C_{38}H_{48}O_{26}$	757, 477, 347	0.2
**5**	Flavonoid	757.1839	757.1833	$C_{32}H_{38}O_{21}$	595, 300	0.8
**6**	Flavonoid	757.1829	757.1833	$C_{32}H_{38}O_{21}$	625, 300	0.5
**7**	Flavonoid	595.1305	595.1305	$C_{26}H_{28}O_{16}$	300	0.0
**8**	Flavonoid	946.2240	946.2259	$C_{42}H_{45}NO_{24}$	799, 595, 300	2.0
**9**	Flavonoid	741.1874	741.1884	$C_{32}H_{38}O_{20}$	285	1.3
**10**	Flavonoid	579.1347	579.1355	$C_{26}H_{28}O_{15}$	284	1.4
**11**	Saponins	1089.5098<br>1059.4971	1089.5123<br>1059.4959	$C_{52}H_{82}O_{24}$<br>$C_{58}H_{75}O_{18}$		2.4<br>1.2
**12**	Saponins	1073.5166	1073.5174	$C_{52}H_{82}O_{23}$		0.7

**13**	Saponins	1059.5021<br>1073.5180<br>1089.5112	1059.5018<br>1073.5174<br>1089.5123	$C_{51}H_{80}O_{23}$<br>$C_{52}H_{82}O_{23}$<br>$C_{52}H_{81}O_{24}$	0.3<br>2.7<br>1.1
**14**	Saponins	1117.5446<br>1087.5307	1117.5436<br>1087.5331	$C_{54}H_{86}O_{24}$<br>$C_{53}H_{84}O_{23}$	0.8<br>2.2
**15**	Saponins	1129.5402<br>1099.5304	1129.5436<br>1099.5331	$C_{55}H_{86}O_{24}$<br>$C_{54}H_{84}O_{23}$	3.1<br>2.4
**16/17**	Saponins	1129.5435<br>1099.5308	1129.5436<br>1099.5331	$C_{55}H_{86}O_{24}$<br>$C_{54}H_{84}O_{23}$	0.1<br>2.3
**18**	Saponins	1131.5579<br>1101.5469	1131.5593<br>1101.5487	$C_{55}H_{88}O_{24}$<br>$C_{54}H_{86}O_{23}$	1.2<br>1.6
**19**	Saponins	1129.5426<br>1099.5340	1129.5436<br>1099.5331	$C_{55}H_{86}O_{24}$<br>$C_{54}H_{84}O_{23}$	0.9<br>0.8
**20**	Saponins	1129.5436<br>1113.5467	1129.5436<br>1113.5487	$C_{55}H_{86}O_{24}$<br>$C_{55}H_{86}O_{23}$	0.0<br>1.8

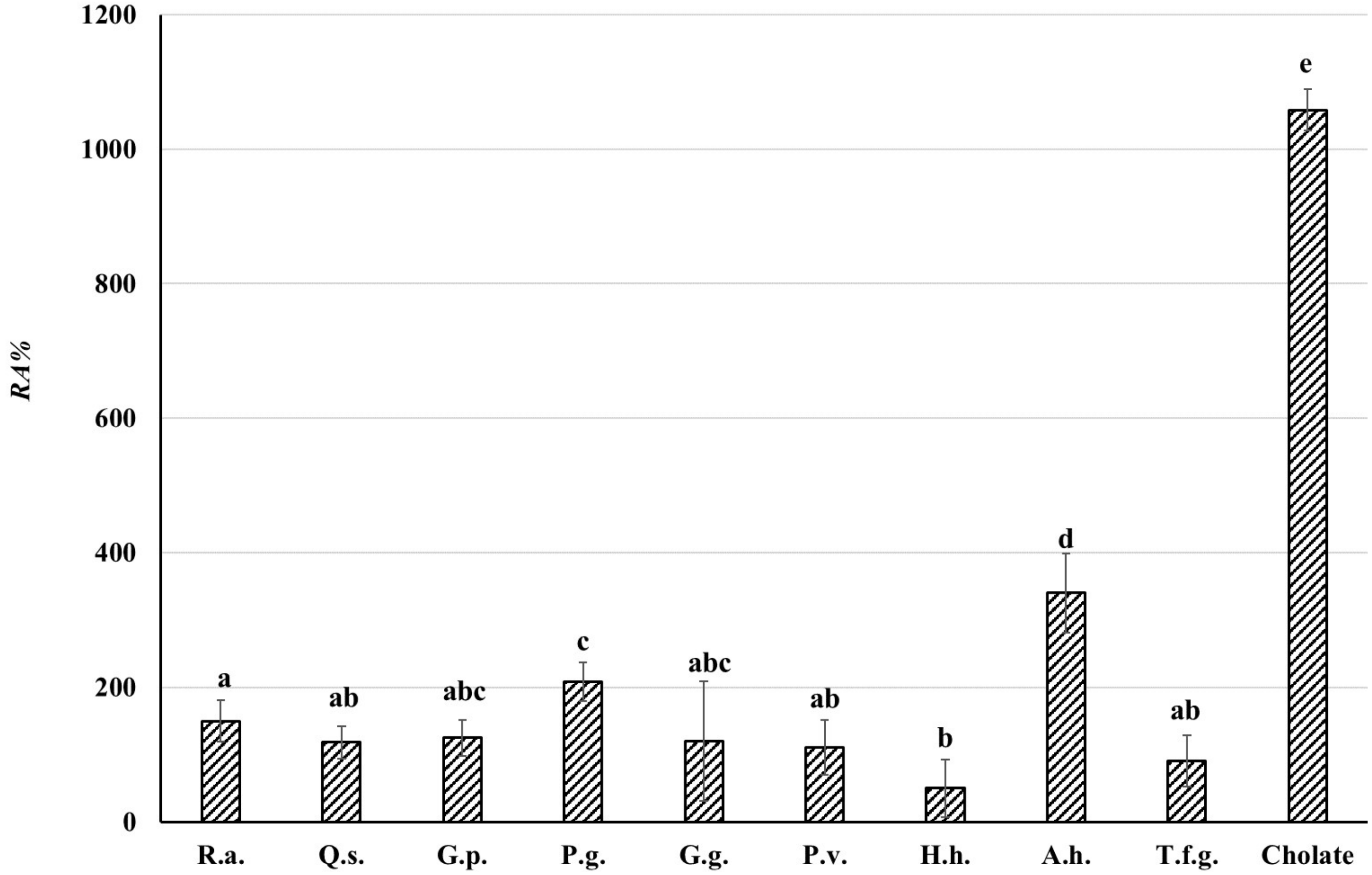

Figure S1 Effect of extracts on lipolytic activity of pancreatin expressed as *RA%* which is the relative activity of the sample compared to the control test (without extract). There is no statistical significance of differences between bars marked with the same letter.

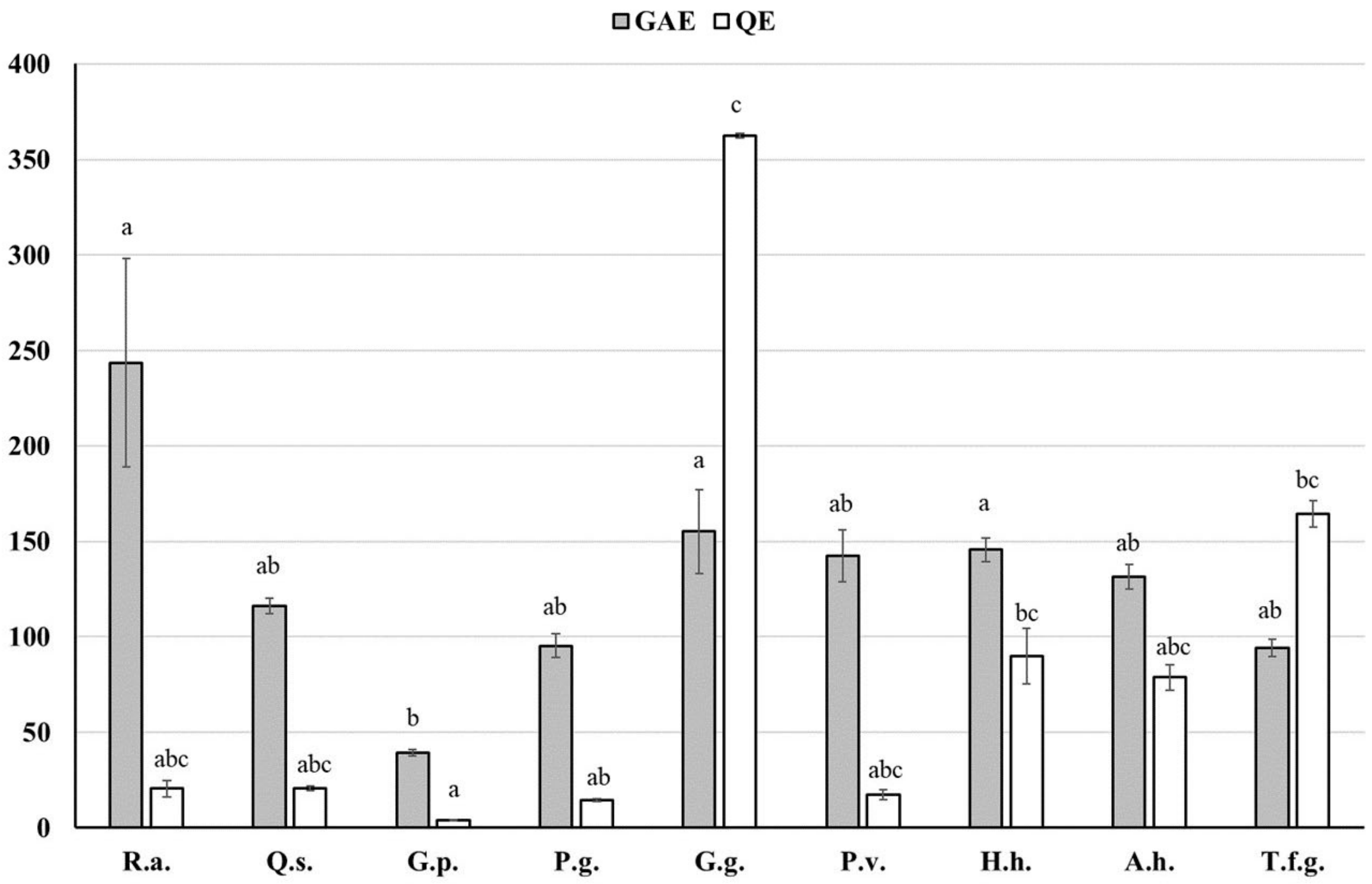

Figure S2 The amount of total phenols and flavonoids in extracts, expressed as gallic acid equivalents (GAE) and quercetin equivalents (QE). There is no statistical significance of differences between bars marked with the same letter.

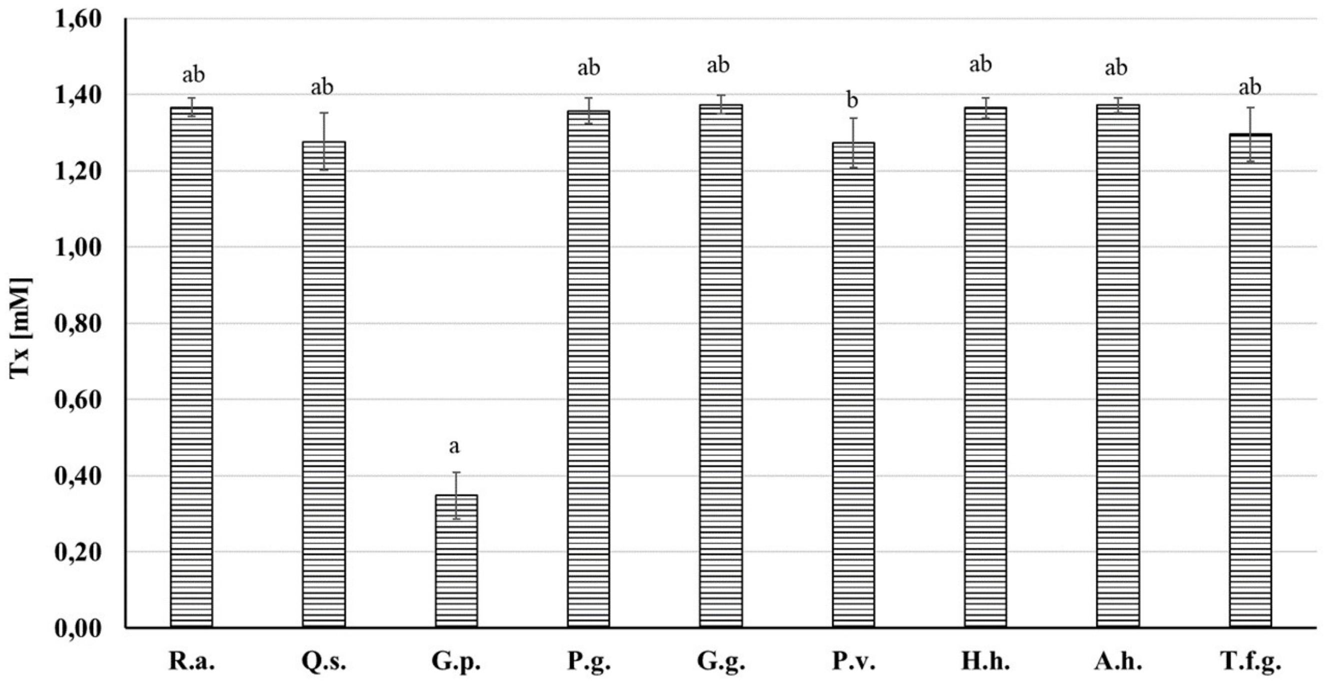

Figure S3 Antiradical activity of extracts measured using ABTS$^{+\bullet}$ cation radical expressed as Trolox equivalents Tx[mM]. There is no statistical significance of differences between bars marked with the same letter.

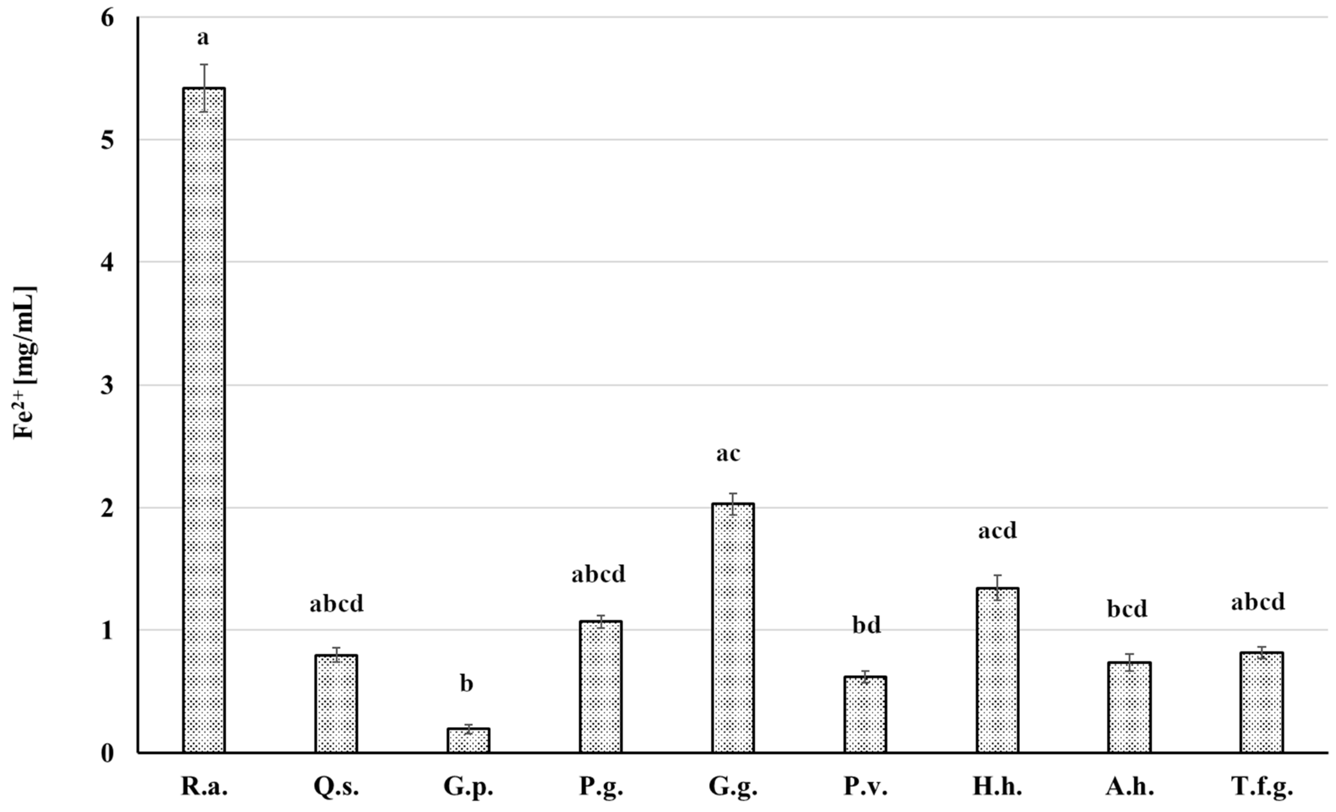

Figure S4 Antiradical activity of extracts presented as concentration of Fe^{2+} in mL of sample Fe2+ [mg/mL. There is no statistical significance of differences between bars marked with the same letter.

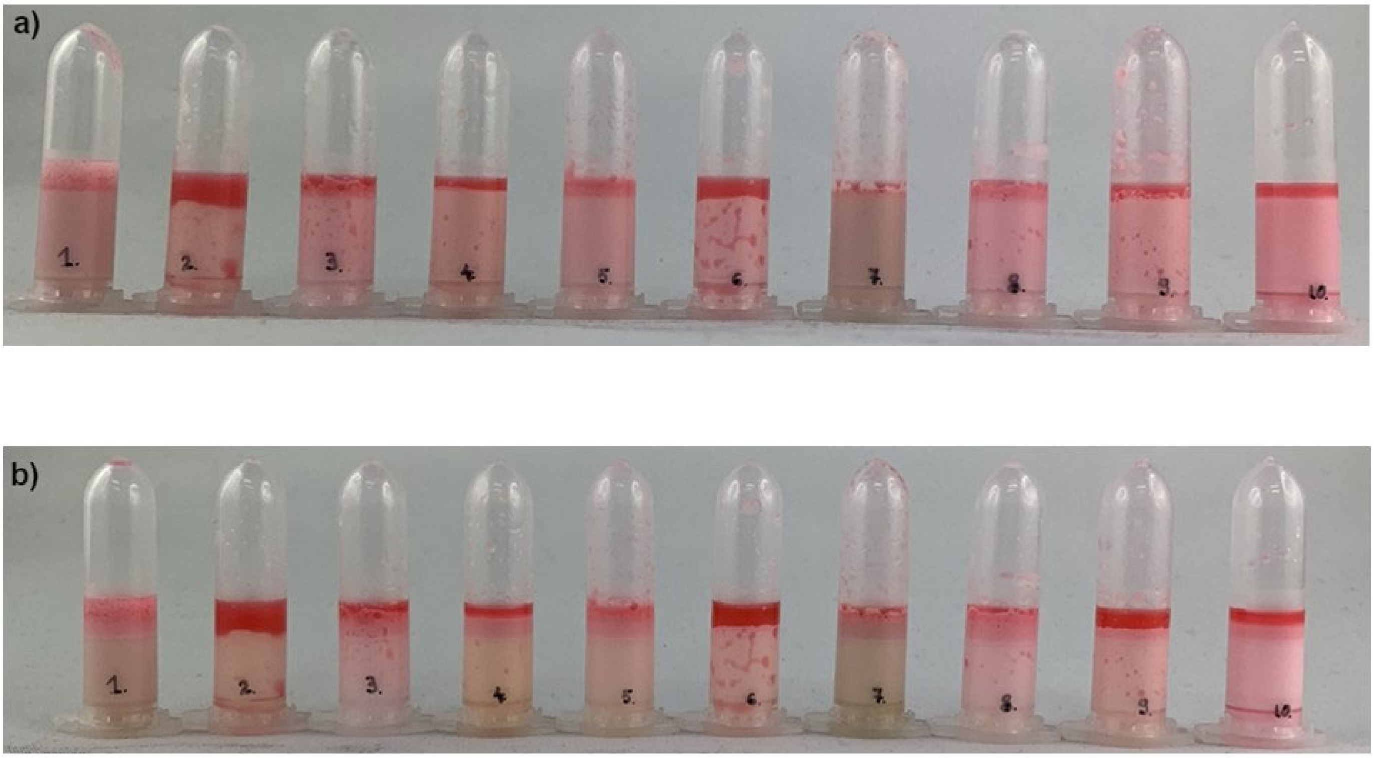

Figure S5 Photographs of emulsions prepared from 1% extract aqueous solutions homogenized with olive oil (stained with Sudan Red IV), 1h after homogenization (a) 24h after homogenization (b). The extracts used as emulsifiers (from left): 1 - *Ruscus aculeatus rhizoma*, 2 - *Quillaja saponaria cortex*, 3 - *Gypsophila paniculata radix*, 4 - *Panax ginseng radix*, 5 - *Glycyrrhiza glabra radix*, 6 - *Primula veris radix*, 7 - *Hedera helix folium*, 8 - *Aesculus hippocastanum semen*, 9 - *Trigonella foenum graecum semen*, 10 - Sodium cholate (positive control for hydrolysis experiments).

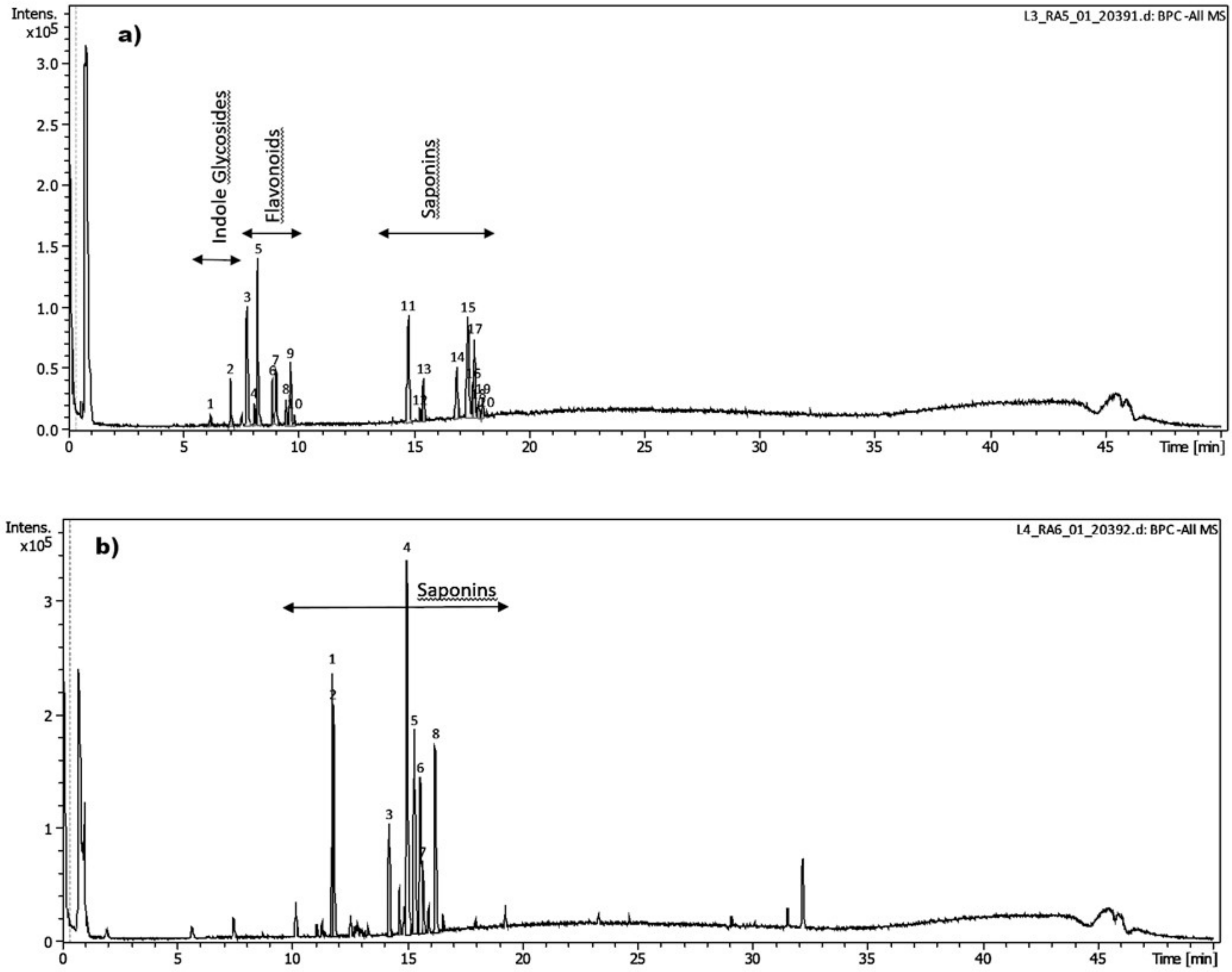

Figure S6 Base peak ion (BPI), chomatogram of the investigated a) *Panax ginseng radix* extracts, b) *Aesculus hippocastani semen* extract.

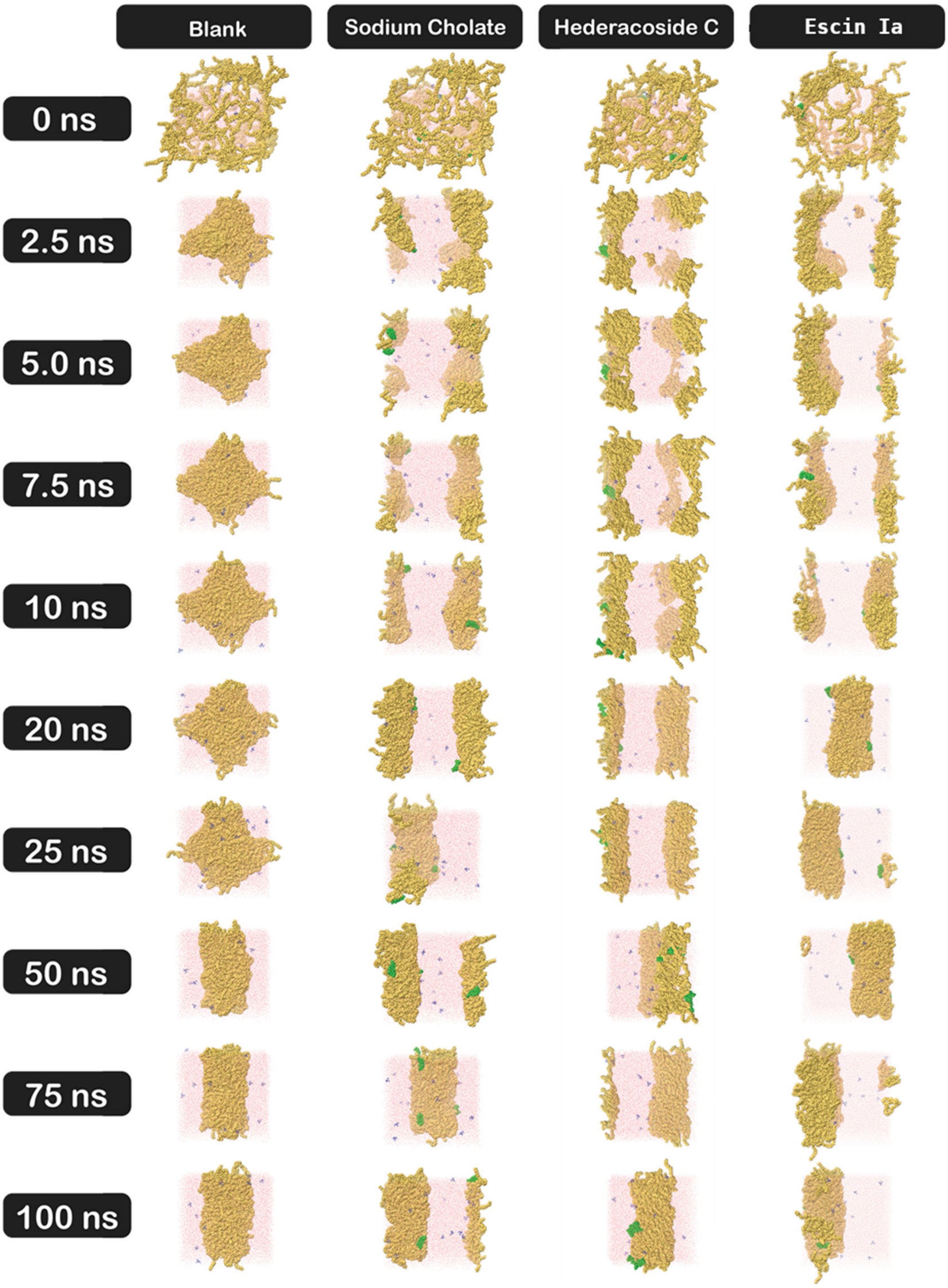

Figure S7 Snapshots from the molecular dynamics simulation of disordered systems.

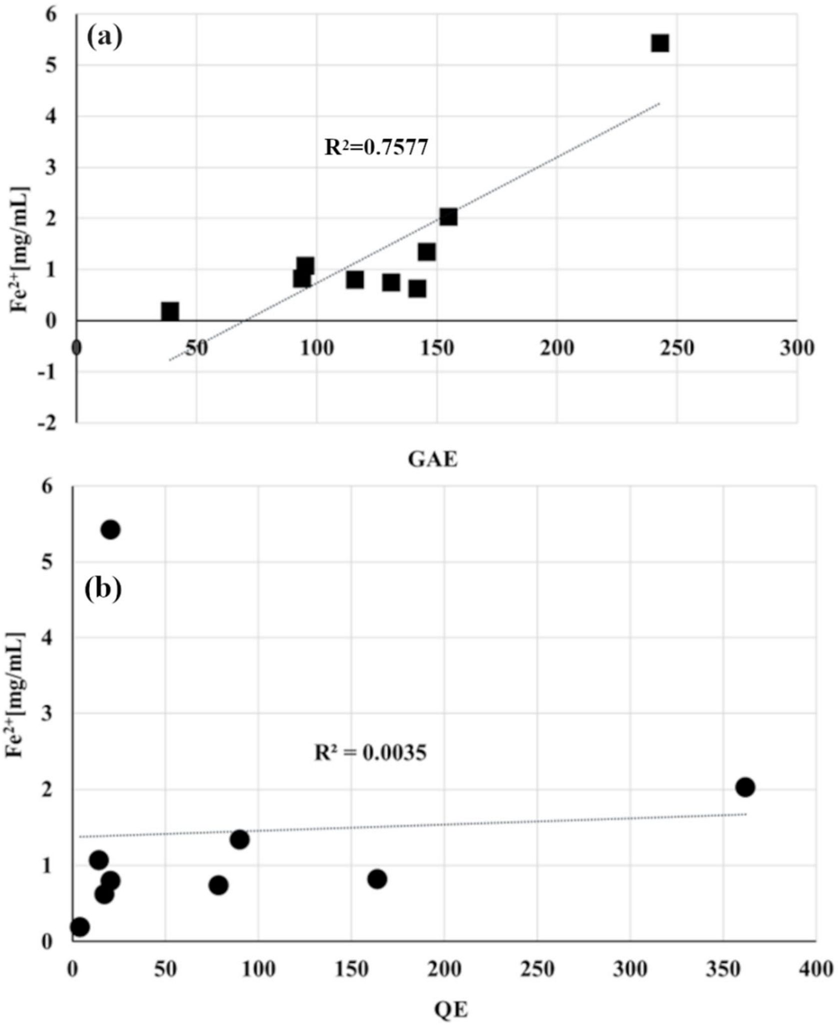

Figure S8 Correlation coefficient between antioxidant activity of extracts and amount of phenolic compounds marked as GAE (a) and flavonoids QE (b).

Video legends:

Video S1. The molecular dynamics simulation of disordered oil/water system.

Video S2. The molecular dynamics simulation of disordered oil/water system with sodium cholate.

Video S3. The molecular dynamics simulation of disordered oil/water system with escin Ia.

Video S4. The molecular dynamics simulation of disordered oil/water system with hederacoside C.
